# The differential view of genotype–phenotype relationships

**DOI:** 10.3389/fgene.2015.00179

**Published:** 2015-05-19

**Authors:** Virginie Orgogozo, Baptiste Morizot, Arnaud Martin

**Affiliations:** ^1^CNRS, UMR 7592, Institut Jacques Monod, Université Paris DiderotParis, France; ^2^Aix Marseille Université, CNRS, CEPERC UMR 7304Aix en Provence, France; ^3^Department of Molecular Cell Biology, University of CaliforniaBerkeley, CA, USA

**Keywords:** genotype, phenotype, genetics, complex trait, GxE, GxG

## Abstract

An integrative view of diversity and singularity in the living world requires a better understanding of the intricate link between genotypes and phenotypes. Here we re-emphasize the old standpoint that the genotype–phenotype (GP) relationship is best viewed as a connection between two differences, one at the genetic level and one at the phenotypic level. As of today, predominant thinking in biology research is that multiple genes interact with multiple environmental variables (such as abiotic factors, culture, or symbionts) to produce the phenotype. Often, the problem of linking genotypes and phenotypes is framed in terms of genotype and phenotype maps, and such graphical representations implicitly bring us away from the differential view of GP relationships. Here we show that the differential view of GP relationships is a useful explanatory framework in the context of pervasive pleiotropy, epistasis, and environmental effects. In such cases, it is relevant to view GP relationships as differences embedded into differences. Thinking in terms of differences clarifies the comparison between environmental and genetic effects on phenotypes and helps to further understand the connection between genotypes and phenotypes.

## Introduction

We sometimes seem to have forgotten that the original question in genetics was not what makes a protein but rather ‘what makes a dog a dog, a man a man.’

(Noble, 2006)

One fundamental question in biology is to understand what makes individuals, populations, and species different from each other. The concept of *phenotype*, which corresponds to the observable attributes of an individual, was coined in opposition to the *genotype*, the inherited material transmitted by gametes. Since the early proposal that genotypes and phenotypes form two fundamentally different levels of biological abstraction (Johannsen, 1911), the challenge has been to understand how they articulate with each other, how genotypes map onto phenotypes. In the last 15 years, more than 1,000 examples of DNA sequence changes have been linked to naturally occurring non-deleterious phenotypic differences between individuals or species in Eukaryotes (Martin and Orgogozo, 2013b). In human, the OMIM^®^ catalog (Online Mendelian Inheritance in Man, http://omim.org/) compiling the genetic determinants of disease-related phenotypes totals more than 4,300 entries and a total of 2,493 published Genome-Wide Association Studies (GWAS) have been uncovering a wealth of sites in the genome that are statistically associated to complex traits (Welter et al., 2014). As the detection of causal links between genetic and phenotypic variation is accelerating, a reexamination of our conceptual tools may help us in finding unifying principles within the swarm of data. Here we reflect on the relationship between genotypes and phenotypes and we address this essay to biologists who are willing to try to challenge their current understanding of phenotypes. We single out one useful point of view, the differential view. We then show that this simple framework remains insightful in the context of pervasive pleiotropy, epistasis, and environmental effects.

## Genes as Difference Makers

**FIGURE 1:**
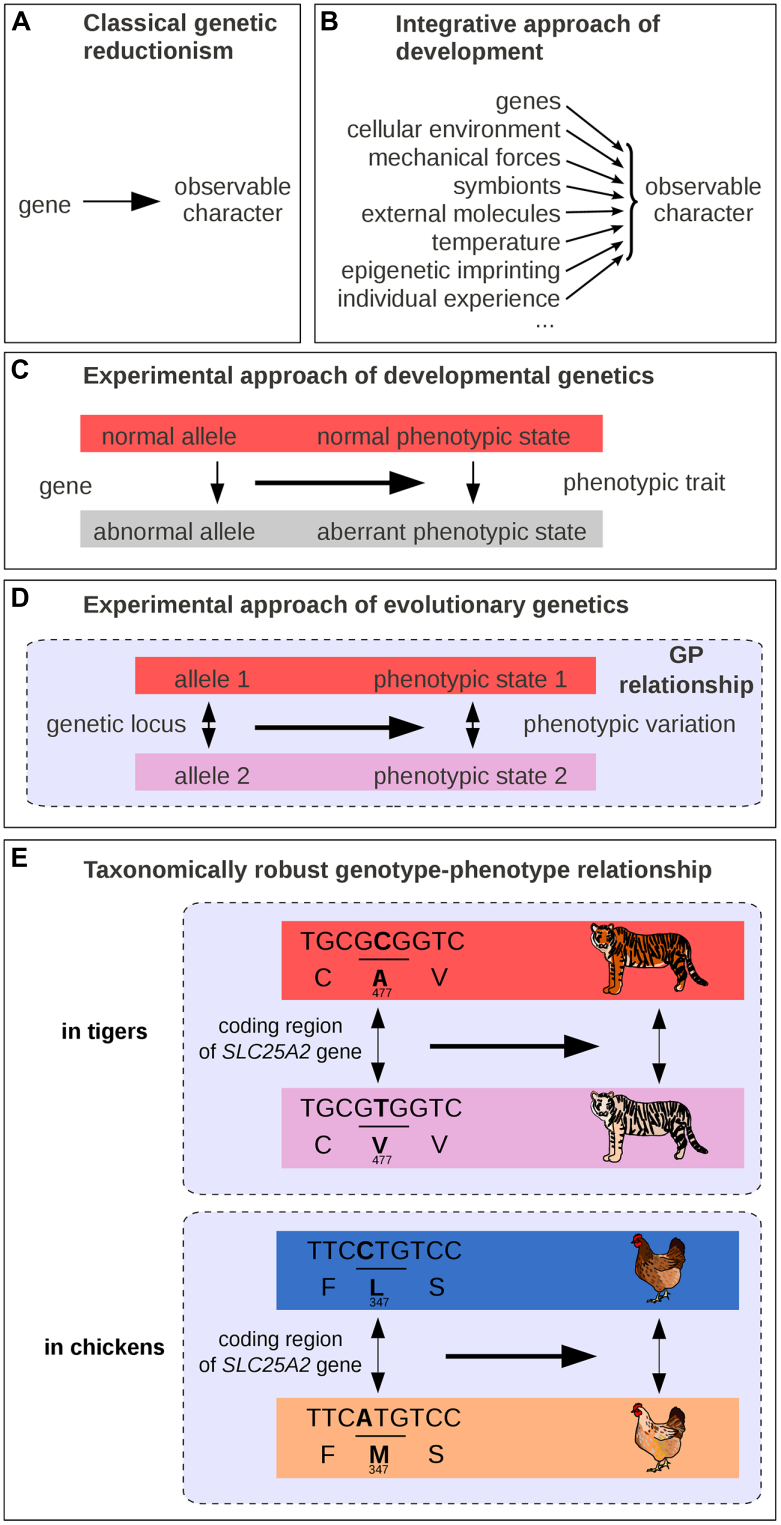
**Schematic representations of GP links. (A)** Traditional representation in classical genetic reductionism. **(B)** Integrative view of developmental biology. **(C)** Scheme of the experimental approach in genetics. **(D)** Scheme of the experimental approach in evolutionary genetics. **(E)** One example of taxonomically robust GP relationship: *SLC45A2* and intraspecific differences in pigmentation in tigers and in chickens. GP relationships are indicated by dashed lines in panels **(D–E)**.

Mutations isolated from laboratory strains have been instrumental to the understanding of the GP map. Under the classical scheme, a mutation is compared to a wild-type reference, and its phenotypic effects are used to infer gene function. This framework often leads to a semantic shortcut: from a genetic change causing a *variation* in phenotype, it is often convenient to assimilate the corresponding gene as a causal determinant of a trait (Keller, 2010; **Figure 1A**). It is common to find headlines expressing these simplifications, trumpeting to wide audiences the discovery of the “longevity” or “well-being” gene, that sacrifice scientific accuracy to psychological impact. Along these lines, should a gene whose mutation is lethal be called a “life gene”? What these over-simplified formulations truly mean is that *variation* at a given gene causes *variation* in a given phenotype (Dawkins, 1982; Schwartz, 2000; Waters, 2007). In fact, a gene alone can neither cause an observable phenotypic trait, nor can it be necessary and sufficient to the emergence of observable characteristics. Genes need a cellular environment, the combined action of multiple other genes, as well as certain physico-chemical conditions to have an observable effect on organisms (**Figure 1B**). For example, brown hair pigmentation in one human being is not just a product of the genes coding for pigment synthesizing enzymes but also of the presence of cells producing pigments of relevant substrate molecules (such as tyrosine for melanin), and of the amount of received sun light (Liu et al., 2013). Thus, the genetic reductionist approach, which only explores a few genetic parameters among the variety of causal factors, is vain to fully address the broad question of what makes hair brown, of what brings forth a particular biological structure, or process in its entirety. Nevertheless, genetic reductionism can be perfectly appropriate for identifying genetic loci where a change causes a phenotypic difference (**Figure 1C**). A *difference* in hair color between two individuals could be due in some cases to their genetic difference. We note, however, that not all phenotypic changes can be attributed to genetic changes. A difference in hair color could also be caused by non-genetic factors such as age, intensity of solar radiation or hair dyeing, or by a combination of both genetic and non-genetic differences.

While modern genetics was in its infancy, Alfred Sturtevant formulated the question of the GP map in simple terms: “one of the central problems of biology is that of differentiation – how does an egg develop into a complex many-celled organism? That is, of course, the traditional major problem of embryology; but it also appears in genetics in the form of the question, How do genes produce their effects?” (Sturtevant, 1932). For long some geneticists may have thought that they were dissecting the morphogenetic mechanisms underlying the *formation* of phenotypic traits, while their experimental approach were in fact uncovering genes whose absence or alteration (mutations, deletions, duplications, rearrangements, etc.) leads to phenotypic *differences* (compare **Figure 1A** with **Figure 1C**). In fact, the sentence “your hair is brown” can be interpreted either as an absolute observation (a description of a particular assemblage of molecules containing defined levels of the dark pigment eumelanin and of the pale pigment pheomelanin) or with implicit reference to other possibilities (it is brown and not of another color). Misconceptions arise because phenotypes are usually defined relative to possibilities that are not formulated explicitly. Our minds and our language often tend to confuse the objects whose variation is under consideration with the variation itself (Keller, 2010), and it is essential to remind that, in genetics, the objects of interest (e.g., a given genotype, an allele or a phenotype) deserve to be defined *relatively* to another reference state.

In summary, the classical genetic reductionist approach is inherently unable to elucidate all the factors responsible for observable characteristics in the living world (Stotz, 2012) but is a powerful and relevant method for dissecting the genetic levers of heritable phenotypic variation. Focusing on phenotypic variation between individuals rather than on absolute characters present in single organisms is key to better comprehend the genetic causes of phenotypic diversity.

## The GP Relationship is between Two Levels of Variation

Thinking in terms of differences makes apparent an abstract entity that encapsulates both genetic and phenotypic levels. This entity is composed of a variation at a genetic *locus* (two alleles), its associated phenotypic change (two distinct phenotypic states), and their relationships (**Figure 1D**). The three of us name the assemblage of these elements a “gephe,” but here we simply call it a “genotype–phenotype relationship” (GP relationship). We will show that the GP relationship is much more than a simple and loosely defined interaction between two levels of organization: it is a cause-and-effect connection that facilitates our understanding of phenotypic diversity.

### The Genetic Part of a GP Relationship

In current genome annotation databases, a *gene* is usually defined as a stretch of nucleic acids that is transcribed and codes for an RNA or a polypeptide with a known or presumed function (Gerstein et al., 2007). The genetic locus underlying a phenotypic difference is not necessarily a gene in the strict sense; it can span a particular base-pair, a coding region, a *cis-*regulatory region, or extend to an entire gene with its *cis*-regulatory regions, or even to a gene cluster (**Table 1**). As previously noted by others (Falk, 1984; Gilbert, 2000; Stern, 2000; Moss, 2003; Griffiths and Stotz, 2013), the concept of gene in developmental biology and in current genome annotation databases is distinct from the concept of gene in evolutionary biology. Here the emphasis is not on the gene itself as defined in genome databases, but rather on a case-by-case functional partitioning of the genome into difference-making loci. The genotypic part of a GP relationship can take the form of various alleles: distinct codons coding for different amino acids, insertions/deletions within a protein coding sequence, diverging versions of a particular *cis-*regulatory element, presence/absence of transposon insertions, number of gene copies within a gene cluster prone to structural variation, etc. Within a genome not all nucleotide sites are associated with phenotypic variation. For instance, there are probably fragments of nucleotide sequences, including the so-called junk DNA (Graur et al., 2015), whose presence does not have any consequence on observable characteristics of the organism, besides being replicated, and possibly transcribed. There are also genetic loci that may have been associated with phenotypic variation in the past and that are no longer associated with phenotypic variation. For example, genetic variation in histone DNA binding coding regions may have been important during the early evolution of eukaryotic cells, but these genetic loci no longer harbor phenotypically relevant variation besides lethal mutations. Within a genome, there are thus nucleotide sites that are absolutely required for life, but that do not harbor viable phenotypically relevant variation themselves.

**Table 1:** A few examples of GP relationships.

Genetic locus	Phenotypic trait	Organisms
Two coding sites in the *ABO HBGG* gene	A/B blood group	Human, chimpanzee, gibbon
*Cis*-regulatory element in the *lactase* gene	Ability to digest milk	Various human populations
Number of duplications of the amylase genes *AMY1* or *AMY2B*	Ability to digest starch	Human, dog
Presence/absence of a complex of adjacent genes coding for carotenoid desaturases and carotenoid cyclase/synthases	Ability to produce carotenoids	Pea aphid, spider mite, gall midge fly, nematode
Coding sites in *opsin* genes	Color vision	Human, cetaceans, fishes, butterflies
Coding region of *F 3’5’H*	Flower pigmentation	Soybean, pea, annual phlox, potato, *Iochroma*
Coding region of *FRIGIDA*	Flowering time	Thale cress, oilseed rape
Coding region of *BADH2*	Fragrance	Soybean, rice
Coding region of *HMA3*	Heavy metal tolerance	Thale cress, rice
Coding and *cis*-regulatory regions of *myostatin*	Muscle size	Cattle, sheep, dog, pig, horse, human
*Cis*-regulatory regions in the *achaete-scute* complex	Number and position of sensory bristles	Fruitflies
*Cis*-regulatory element in the *pitx1* gene	Pelvis morphology	Stickleback fish
Coding region of *SLC45A2*	Pigmentation of eye, hair, and skin	Human, tiger, chicken
Coding region of *Mc1R*	Pigmentation of hair and skin, but not eye	Human, mouse, cattle, chicken, guinea pig, horse, fox, pig, sheep, dog, rabbit, bear, jaguar, jaguarundi, squirrel, birds, sand lizard
*Cis*-regulatory regions of *tan* and *ebony*	Pigmentation pattern	Fruitflies
*Cis*-regulatory elements in the *shavenbaby* gene	Position and number of trichomes	Fruitflies
*Cis*-regulatory elements in the *optix* gene	Red color pattern on butterfly wings	Longwing butterflies
Coding region of *hemoglobin alpha* and *beta* chain genes	Resistance to hypoxia	Human, llama, crocodile, deer mouse, waterfowl
Coding region of *Ace*	Resistance to organophosphate insecticides	Potato beetle, aphids, mosquitoes, house fly, fruit flies, oriental fruitfly
Presence/absence of a *CypA* insertion within the *TRIM5a* gene	Resistance to retrovirus	Owl monkey, macaque, Old World monkeys
Coding sites in the *Nav1.4* gene	Resistance to tetrodotoxin or saxotoxin	Snakes, pufferfish, clam
Coding region of *TAS2R38*	Sensitivity to bitterness	Human, chimpanzee
*Cis*-regulatory and coding regions in *Agouti*	Skin and coat pigmentation	Human, deer mouse, cattle, pig, dog, cat, horse, fox, domesticated fox, quail, sheep
Number of duplications of the glucose transporter gene *HXT6*	Survival in low-glucose environment	Yeast
Number of duplications of the *CCL3L1* gene	Susceptibility to HIV infection and progression rate of AIDS after infection	Human
*Cis*-regulatory element in the *WntA* gene	Wing pigmentation pattern	Butterflies

See (Martin and Orgogozo, 2013a) for references and for additional cases.

### The Phenotypic Part of a GP Relationship

The phenotypic counterpart of the GP relationship refers to a kind of variation (hair color, level of toxin resistance, etc.) rather than to a state (blond hair, taster of phenylthiocarbamide, etc.; **Table 1**).

The phenotype associated with a genetic change is not necessarily confined to the organism that harbors the genetic mutation. For example, the difference between left- and right-coiled shells in the snail *Lymnaea peregra* is determined by a single genetic locus with maternal effect: the genotype of the mother, but not of the individual itself, is responsible for the direction of shell coiling (Boycott et al., 1931). In other cases, the causal genetic change lies within symbiont bacteria: aphid thermal tolerance can vary between individuals due to a point mutation in their bacterial symbiont (Dunbar et al., 2007). Certain phenotypic effects can also come up at a level higher than the organism harboring the genetic change (Dawkins, 1982), one exemplary case being the social organization of an ant colony (Wang et al., 2013).

### The Differential Part of a GP Relationship

As defined above, the GP relationship encompasses a genetic difference and a phenotypic difference. The relationship of *difference* at both the genetic and the phenotypic level is quite abstract, and it can correspond to three distinct differences within the living world: (#1) a difference between two reproductively isolated taxa (living or extinct), (#2) a difference segregating within a population, and (#3) the difference that first appeared during evolution, between an organism harboring the ancestral allele/trait and its direct descendant which evolved the new allele/trait. Of note, the variation in phenotype does not always immediately follow the emergence of the new causing mutation, but can appear later from the singular assortment of alleles that are segregating in the population. For example, a new phenotype of reduced armor plates appeared in a freshwater stickleback population when a recessive *EDA* allele already present at cryptic levels ended up in a homozygous state in one individual (Colosimo et al., 2005; Jones et al., 2012). A major conceptual advance made by Charles Darwin was to relate variation among individuals within an interbreeding group (difference #2) with variation between taxonomic groups in space and time (difference #1; Lewontin, 1974a).

Note also that certain phenotypic changes may appear at the level of the entire organism when the “causative” mutation is accompanied by additional somatic mutations that are highly likely. For example, in women carrying a wild-type allele and a mutant allele *of BRCA1*, cells can produce wild-type *BRCA1* proteins since they carry one copy of the wild-type *BRCA1* allele. Nevertheless, these women have up to an 80% risk of developing breast or ovarian cancer by age 70 compared to women carrying two wild-type copies of *BRCA1*, due to the appearance of additional deleterious mutations within the wild-type *BRCA1* allele in their somatic breast cells (Narod and Foulkes, 2004).

Importantly, the GP difference is always defined relative to a population, or taxon, of interest (Sober, 1988). In less medically developed countries, humans carrying two defective copies of the phenylalanine hydroxylase gene have serious medical problems including seizure and intellectual disabilities. In contrast, in most developed countries, such humans are diagnosed at birth and have a normal life span with normal mental development thanks to a phenylalanine-restricted diet (Armstrong and Tyler, 1955). Therefore the GP relationship involving the phenylalanine hydroxylase defective mutation is context-dependent: the mutation is associated with health problems in less medically developed countries but not in other countries. This example shows that the causal relationship between a genetic change and its associated phenotypic change can hide multiple embedded parameters (such as medical practices for the phenylalanine hydroxylase case) within the *ceteris paribus* assumption of “all other things being equal.”

In summary, the GP relationship is best viewed as a relationship between two variations, one at the genotypic level, and one at the phenotypic level. The human mind can elaborate concepts of increasing abstraction: concepts of things (e.g., a cell), concepts of change (e.g., evolution), and concepts of relations (e.g., homology; Cassirer, 1910; Simondon, 1968). Here the concept of GP relationship establishes a relation between two *changes* (genetic and phenotypic). In the next paragraphs we will show that, compared to the usage of intuitive concepts of things, this detour through increased abstraction may prove more efficient to better understand phenotypic diversity.

## Several Current Representations of the Connection between Genotype and Phenotype Implicitly Dismiss the Differential View

We argued above that the differential view should always be kept in mind when thinking about the connection between genotypes and phenotypes. GWAS, which represent the most popular method to detect genomic loci that are associated with complex traits in populations, are based on the analysis of differences (Visscher et al., 2012). Nevertheless, in current research the differential view is sometimes implicitly dismissed. When multiple factors are observed to influence phenotypic traits (**Figure 1B**), the differential view is considered as too simplistic and researchers often prefer to focus back on phenotypes of single individuals, without explicitly relating them to a phenotypic reference.

**FIGURE 2:**
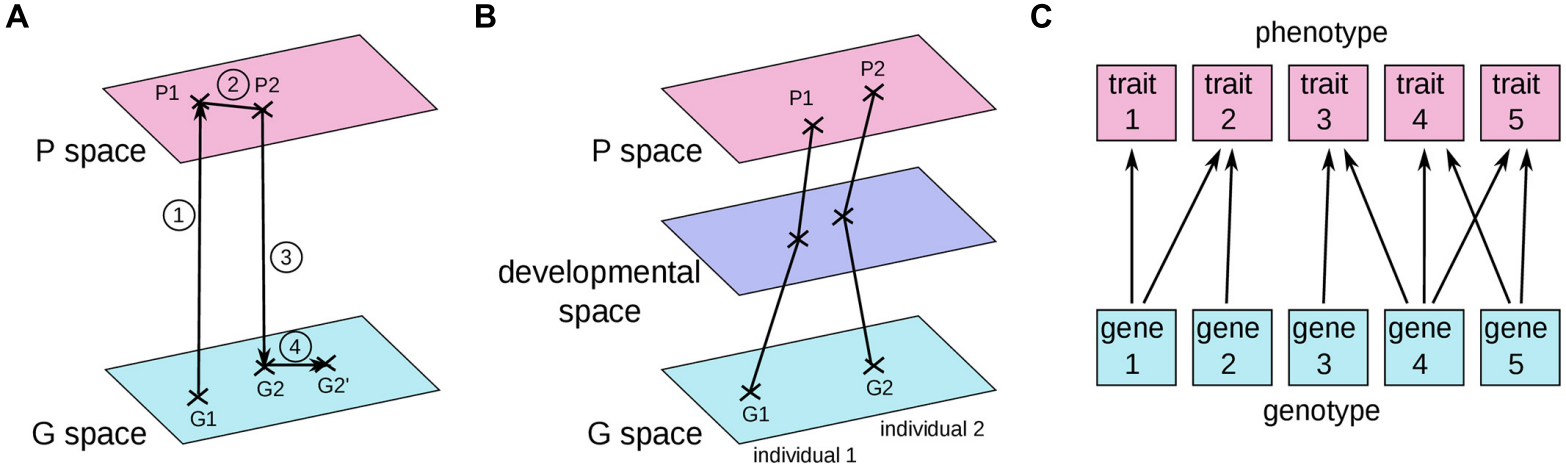
**Three current graphical representations of GP maps. (A)** The early version of the GP map proposed by Lewontin (1974a). **(B)** A GP map where each point represents a single individual (Houle et al., 2010; Gjuvsland et al., 2013; Salazar-Ciudad and Marín-Riera, 2013). **(C)** The relationships between traits and genes, as depicted by Wagner (1996). See text for details.

In most current articles, the problem of connecting the genotype to the phenotype is framed in terms of genotype and phenotype maps. The first GP map was introduced by Richard Lewontin in his book “The genetic basis of evolutionary change” (Lewontin, 1974a; **Figure 2A**). He indicated the average genotype of a population as a point in the space of all possible genotypes (G space) and the average phenotype of the same population as a corresponding point in the space of all possible phenotypes (P space). The evolutionary process was thus decomposed into four steps: (1) the average phenotype is derived from the development of the distinct genotypes in various environments; (2) migration, mating, and natural selection acts in P space to change the average phenotype of the initial population into the average phenotype of the individuals which will have progeny; (3) the identity of successful parents determines which genotypes are preserved; and (4) genetic processes such as mutation and recombination modify position in G space.

In another common graphical representation (**Figure 2B**), a point in the G space and its corresponding point in the P space correspond to the genotype and the phenotype of a single individual (Fontana, 2002; Landry and Rifkin, 2012). Under such a representation, the abstract object that we defined above as the GP relationship would correspond to a “move” in genotype space associated with a “move” in phenotype space (or, better, a sum of several “moves” in genotype, and phenotype spaces because several distinct genomes can carry the two alternative alleles of a given GP relationship). In a third representation put forward by Wagner (1996; **Figure 2C**), individual genes are connected to individual traits.

Although these three graphical representations of GP maps may facilitate our understanding of certain aspects of biology, in all of them the GP relationship and the differential view are not easy to grasp. It is quite perplexing that the first person to draw such a GP map was Richard Lewontin, an eloquent advocate of the differential view (see for example his preface to Oyama, 2000, a masterpiece of persuasion). Because these graphics focus on individual rather than differential objects, we believe that these three representations implicitly incite us to go back to the more intuitive idea of one genotype associated with one phenotype. Losing sight of the differential view might also come from the molecular biology perspective, where proteins are viewed as having causal effects on their own, such as phosphorylation of a substrate or binding to a DNA sequence. Because of the two entangled definitions of the gene, either as encoding a protein, or as causing a phenotypic change (Griffiths and Stotz, 2013), it is easy to move from a differential view to a non-differential view of the GP relationship.

In summary, many current mental representations of the connection between genotype and phenotype implicitly dismiss the differential view. We will now show that the differential view is compatible with the fact that phenotypic traits are influenced by a complex combination of multiple factors and that we can find a relevant schematic representation of GP relationships.

## The Problem of Pleiotropy

Decomposing an organism into elementary units such as anatomical structures has been instrumental in many biology disciplines such as physiology, paleontology and evolution. However, the issue is to identify the decomposition into characters that is most adequate for the question of interest. For questions related to relationships between organs of various individuals or species (such as homology), it might be appropriate to keep the traditional decomposition into anatomical structures (Wagner, 2014). Richard Lewontin and Günter Wagner defined characters as elements within an organism that answer to adaptive challenges and that represent quasi-independent units of evolutionary change (Lewontin, 1978; Wagner, 2000). Their definition deals with absolute traits observed in single organisms (for example the shape of a wing, or the number of digits in an individual) and is thus far from the differential view. Here, to better apprehend evolution and phenotypic diversity of the living world, we propose to decompose the observable attributes of an organism into multiple elementary GP variations that have accumulated through multiple generations, starting from an initial state. We insist that under this perspective, characters are not concrete objects (such as skin) but abstract entities defined by the existence of *differences* between two possible observable states (for example skin color). As an analogy, one can imagine two ways to produce a well-worn leather shoe of a particular shape. One can either assemble the different atoms into the same organization, or one can buy a shoe in a store and then subject it to a series of mechanical forces. We are naturally inclined to compare organisms to machines, and to think in terms of pieces that must be assembled to make a functional whole. However, the rampant metaphor of the designer or maker is inadequate for understanding the origin of present-day organisms (Coen, 2012). To understand the phenotypic features of a given organism it is more efficient to decompose it into abstract changes that occurred successively across evolutionary time, and not across developmental time. The initial state is a hypothetical ancestor of the organism under study.

Certain mutations (qualified as pleiotropic) are observed to affect several organs at once while others alter only one at a time (Paaby and Rockman, 2013; Zhang and Wagner, 2013). For pleiotropic mutations, we consider that the GP relationship should include all the phenotypic changes (in diverse organs, at various stages, etc.) associated with the genetic difference. For instance, the V370A mutation of the EDAR receptor is associated not only to hair thickness but also to changes in sweat gland and mammary gland density in Asian populations (Kamberov et al., 2013). The GP relationship is, in such cases, one-to-multi. Considering skin and eye as independent anatomical modules of the human body might seem appropriate for many evolutionary changes, but it is somewhat inadequate in cases where these two organs evolved a new pigmentation trait at once through a single mutation in the *SLC45A2* gene (Liu et al., 2013). Reasoning in terms of GP relationships strikes off the problem of finding a relevant decomposition into elementary anatomical structures. The elementary GP relationships themselves appear as adequate semi-independent modules, whose combination can account for the observable characteristics of an organism.

## The Problem of Continuous Complex Traits

**FIGURE 3:**
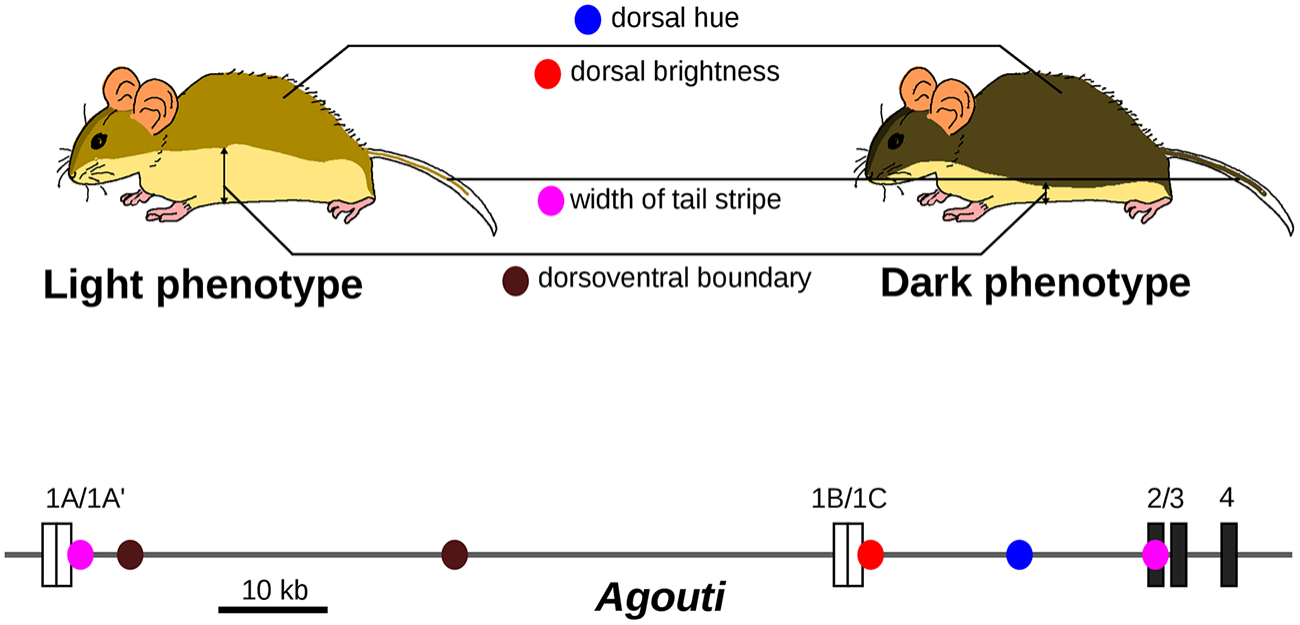
**Evolution of light-colored beach mice is caused by several mutations with distinct pigmentation effects in the *Agouti* locus.** The dark and light phenotypes can be decomposed into four phenotypic traits, which are associated with different single nucleotide polymorphisms (SNPs, colored dots) located in the *Agouti* gene. Only SNPs with inferred selection coefficient of the light allele higher than 0.1 are shown. Coding exons are represented as dark boxes and untranslated exons as white boxes. Adapted from Linnen et al. (2013).

Under the differential concept of GP relationships, one crucial point is to decompose observable traits into a series of semi-independent phenotypic variations, that is to identify the elementary changes that have occurred during evolution. Experimental approaches are available to decompose a given phenotypic difference into appropriate finer sub-variations. For example, crossing plants with different leaf shapes yields a progeny that exhibits a composite range of intermediate leaf shapes. Principal component analysis uncovered elementary leaf shape changes that can together account for the difference in shape between parental lines and that appear to be caused by distinct genomic regions (Langlade et al., 2005). This suggests to some extent that “the sum obscures the parts.” What we traditionally consider as complex traits can be made of simpler traits, more amenable to genetic analysis. Another illuminating example is the abdominal pigmentation in the *Drosophila dunni* group. Taken as a single variable, the levels of pigmentation show a complex genetic architecture, but decomposing adult patterns into anatomical sub-units unravels discrete genetic control for each sub-trait (Hollocher et al., 2000). A better known case is the evolution of body color in beach mice. The difference in color between light-colored beach mice and dark mice can be decomposed into distinct phenotypes (dorsal hue, dorsal brightness, width of tail stripe, and dorsoventral boundary), which are all associated with distinct mutations in the *Agouti* gene (Linnen et al., 2013; **Figure 3**). Each *Agouti* genetic locus appears to be dedicated to the specification of pigmentation in a given body part. Together, they form a group of tightly linked loci that are associated with changes in coat pigmentation.

While complex traits may not always be reducible to a suite of simple GP relationships, it is possible that traits such as adult human height, the most emblematic quantitative trait predicted to consist of many genetic effects of small size (Fisher, 1930), might also be decomposed into elementary variations, each explaining more discrete sub-traits. While some determinants of human height such as *LIN28B* have been associated to adult height at different ages, other genes have only reached statistical significance in stage-specific studies focusing on fetal growth and height velocity at puberty (Lettre, 2011). In other words, these data suggest that human height may be a composite trait that is modulated by several GP relationships, each acting at different phases of developmental growth.

## The Problem of Epistasis and GxE

Gene-by-Environment (GxE) interaction occurs when the phenotypic effect of a given genetic change depends on environmental parameters. Similarly, epistasis, or GxG interaction, occurs when the phenotypic effect of a given genetic change depends on the allelic state of at least one other locus (Phillips, 2008; Hansen, 2013). There is increasing evidence that GxG and GxE interactions are of fundamental importance to understand evolution and inheritance of complex traits (Gilbert and Epel, 2009; Hansen, 2013). We propose that both phenomena can be integrated into the basic GP differential framework, where both GxG and GxE interactions inject a layer of context-dependence, and result in differences embedded within differences.

**FIGURE 4:**
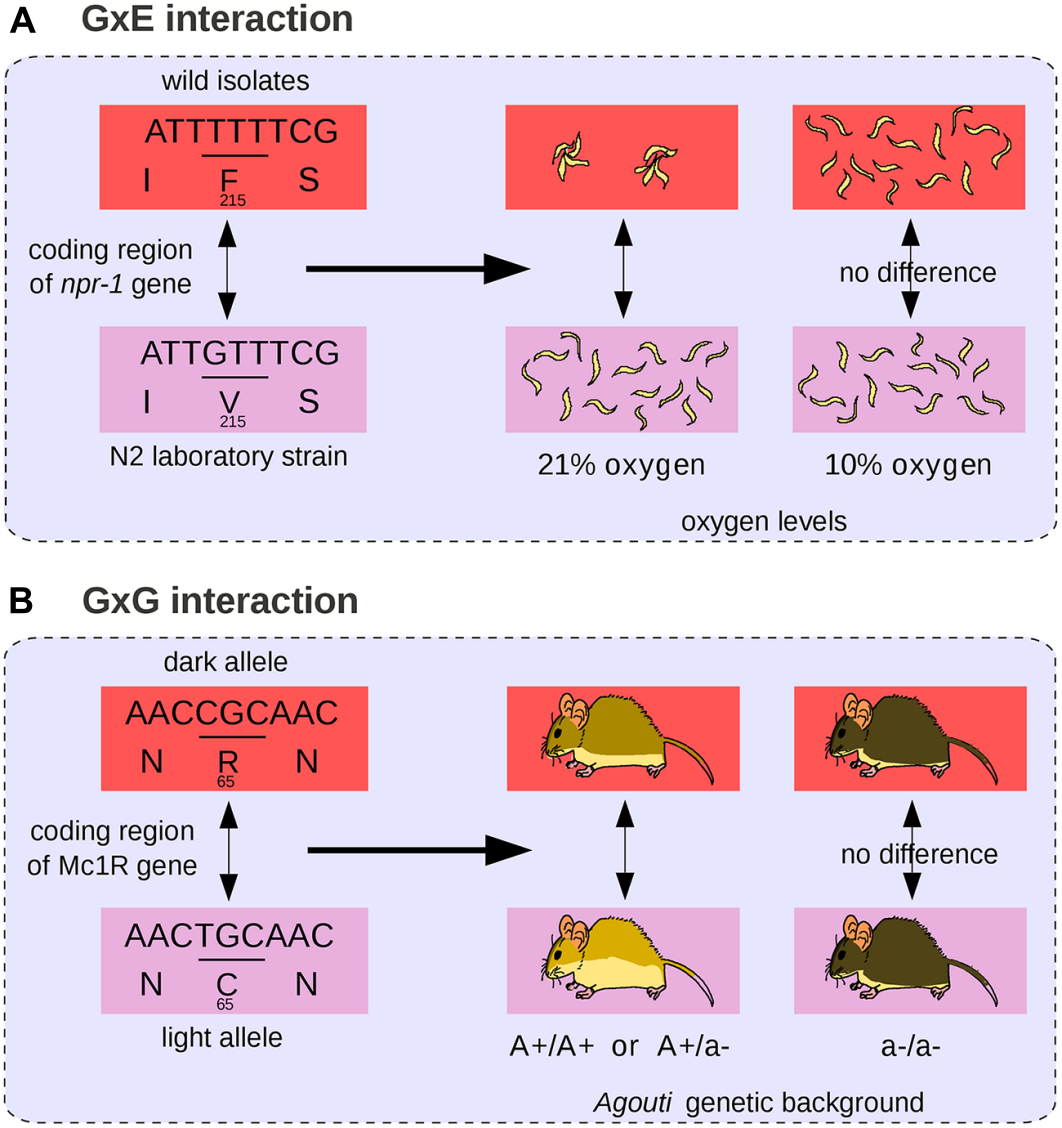
**Gene-by-environment (GxE) and GxG interactions. (A)** The *npr-1* coding mutation affects nematode aggregation behavior at 21% oxygen levels but not at 10% (Andersen et al., 2014). **(B)** The *Mc1R* coding mutation affects mouse body pigmentation in presence of dominant light alleles of *Agouti* but not in an *Agouti* homozygous background for the recessive dark allele (Steiner et al., 2007).

The difference in color pigmentation between dark and light-colored beach mice mentioned previously (**Figure 3**) is not only due to mutations in *Agouti* but also to a coding mutation in the *MC1R* gene that decreases pigmentation (Steiner et al., 2007; **Figure 4B**). The effect of the *MC1R* mutation is visible only in presence of the light-colored-associated derived *Agouti* haplotype. Here the *Mc1R* locus is considered to interact epistatically with the *Agouti* locus. In this case, we propose that the GP relationship does not comprise a single phenotypic difference but two *possible* phenotypic differences (a change in coat pigmentation or no change at all). The choice between these two phenotypic differences is determined by the genetic background (here at the *Agouti* locus). The differential view thus remains relatively straightforward for two-loci interactions: the context-dependence of the phenotype is translated into a choice between two possible phenotypic differences. We propose that a GP relationship involving a mutation subjected to multiple epistatic interactions should comprise all possible phenotypic differences that can result from the mutation in all genetic backgrounds. Among all possible phenotypic variations, the phenotypic difference that will be observed is determined by other genetic loci. In general, GxG interactions involve multiple sites that are dispersed across the genome (Bloom et al., 2013).

An example of GxE interaction (see also **Figure 4A**) is the naturally occurring loss-of-function allele of *brx* in *Arabidopsis* plants, which is associated with accelerated growth and increased fitness in acidic soils, and with severely reduced root growth compared to wild-type in normal soils (Gujas et al., 2012). GxE interactions are usually analyzed in the form of a *norm of reaction*, which represents all the observable traits of a *single* genotype across a range of environments (Johannsen, 1911; Sarkar, 1999). In the case of GxE interactions, we propose that the GP relationship should comprise all the possible phenotypic *changes* that can be caused by the associated genetic change across various experimental conditions. The associated phenotypic change is thus a difference between two norms of reaction. A textbook example is the variation in temperature-size rule in *C. elegans*. Like most other animals, *C. elegans* nematodes grow larger at low temperature, but a wild-type laboratory strain of *C. elegans* originating from Hawaii shows no variation in body size across various temperatures. An amino acid change in a calcium-binding protein is responsible for the decreased ability of the Hawaiian strain to grow larger at low temperature (Kammenga et al., 2007). Here the norm of reaction (representing nematode body size across a range of temperatures) differs between nematodes and the associated GP relationship encompasses the difference between these two slopes.

The range of phenotypic variations embodied within GP relationships subjected to GxG and GxE interactions can be quite overwhelming, especially in cases when several tissues are affected by the same mutation, and when the phenotypic variation of each tissue is influenced by other genomic loci and by environmental conditions. In fact, the phenotypic effects of a mutation always rely on other pieces of DNA from the same genome, so that any GP relationship can be considered to experience epistasis. In other words, a genetic locus affecting a phenotype never acts independently of other DNA sequences. For instance, a given opsin allele will only lead to particular color vision properties if an eye is formed and if this eye receives light during its development, allowing effective vision neural circuits to form. For the differential view to be tractable, we advise not to consider all possible genetic backgrounds and environmental conditions, but to restrict possibilities to potential environments, and segregating alleles that are relevant to the population of interest (Sober, 1988).

In summary, in presence of epistasis or GxE interactions, a genetic change is not associated with a single phenotypic difference but with multiple possible phenotypic differences, among which one will be achieved, depending on the environment and the genetic background. The context-dependence can be represented schematically as GP differences embedded into other genotype and environment differences.

### The Differential View of Genetic and Environmental Effects on Phenotypes

As underlined by multiple authors (most notably Waddington, 1957; Oyama, 2000; Keller, 2010), genes and environment act jointly on the phenotype, and in most cases it is impossible to disentangle the effect of one from the other. Here we show that reasoning in terms of differences helps to clarify the comparison between genetic and environmental effects on phenotypes. However, we identify certain cases where the comparison remains difficult.

**FIGURE 5:**
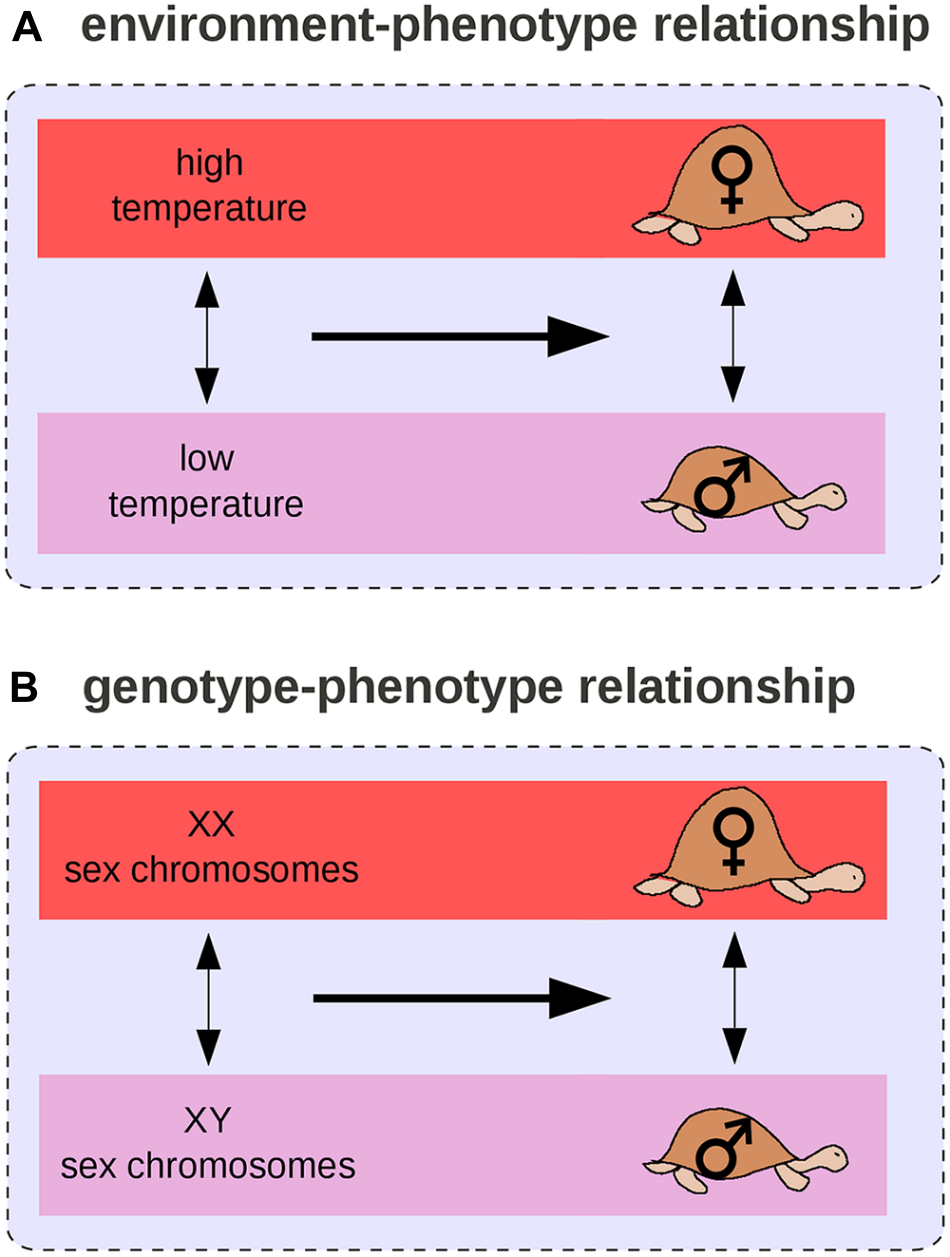
**Environment–phenotype relationship vs. GP relationship for sex determination in turtles. (A)** In some species, the temperature during embryonic development determines the sex of the adult. **(B)** In others, sex is determined by sex chromosomes.

By analogy with the GP relationship, we can define the environment-phenotype relationship as an environmental variation (two environments), its associated phenotypic change (distinct phenotypic states), and their relationships. For example, in many turtle species, a change in temperature during egg development is associated with the male/female sex difference (**Figure 5A**) and at least six transitions from environmental to genetic sex determination (**Figure 5B**) occurred across the turtle phylogeny (Pokorná and Kratochvíl, 2009). In this case, environmental and genetic effects can be compared: sex chromosomes and temperature have the same phenotypic effect on turtles. Such observations led West-Eberhard (2003, 2005) to propose the “genes as followers” hypothesis, which suggests that novel phenotypic states are more likely to arise first from a change in the environment than from a genetic mutation, and that mutations occur only later, in modifying the threshold for expression of the novel trait. West-Eberhard (2003, 2005) extrapolated from differences segregating within populations (difference #2) to differences that arose temporally during the evolution of a population (difference #1).

The independent evolution of directional left–right asymmetry from symmetrical ancestors in multiple lineages has provided a major argument supporting the “gene as follower” hypothesis (Palmer, 2004). Under this framework, it is stipulated that directional asymmetry, where all individuals are same-sided, has often evolved from a “random asymmetry” state, where the directionality will depend on environmental factors and thus vary between genetically identical individuals. For instance, the strongest claw of a lobster will develop based on usage and has a priori equal probabilities to develop on the left or on the right side. We can see how the “genes as followers” formula applies here: the environment triggers an asymmetry, and later in evolution some genetic effects can bias its directionality on one side or the other. But while asymmetry “occurs before genetic variation exists to control it,” the differential view makes it clear that the genetic effect on directionality is not comparable to the environmental effect that triggers the asymmetry. The genetic change makes a switch between the final 100% same-sided condition and an initial condition where 50% of the cases are dextral and 50% sinistral. In contrast, the two alternative phenotypic states resulting from variation in the environment are considered to be 100% dextral and 100% sinistral. This example shows that for the sake of accuracy it is important to explicitly state the differences that are being considered within a GP relationship.

The differential view provides a theoretical framework that can help in designing experiments to investigate the proper variables: one can compare different genotypes in a fixed environment (classic GP relationship), compare the response of a fixed genotype to two different environments (phenotypic plasticity), or compare the sensitivity of two different genotypes to two different environments (wherein the phenotypic variation becomes a *difference in a difference*; see for example Engelman et al., 2009; Thomas, 2010).

**FIGURE 6:**
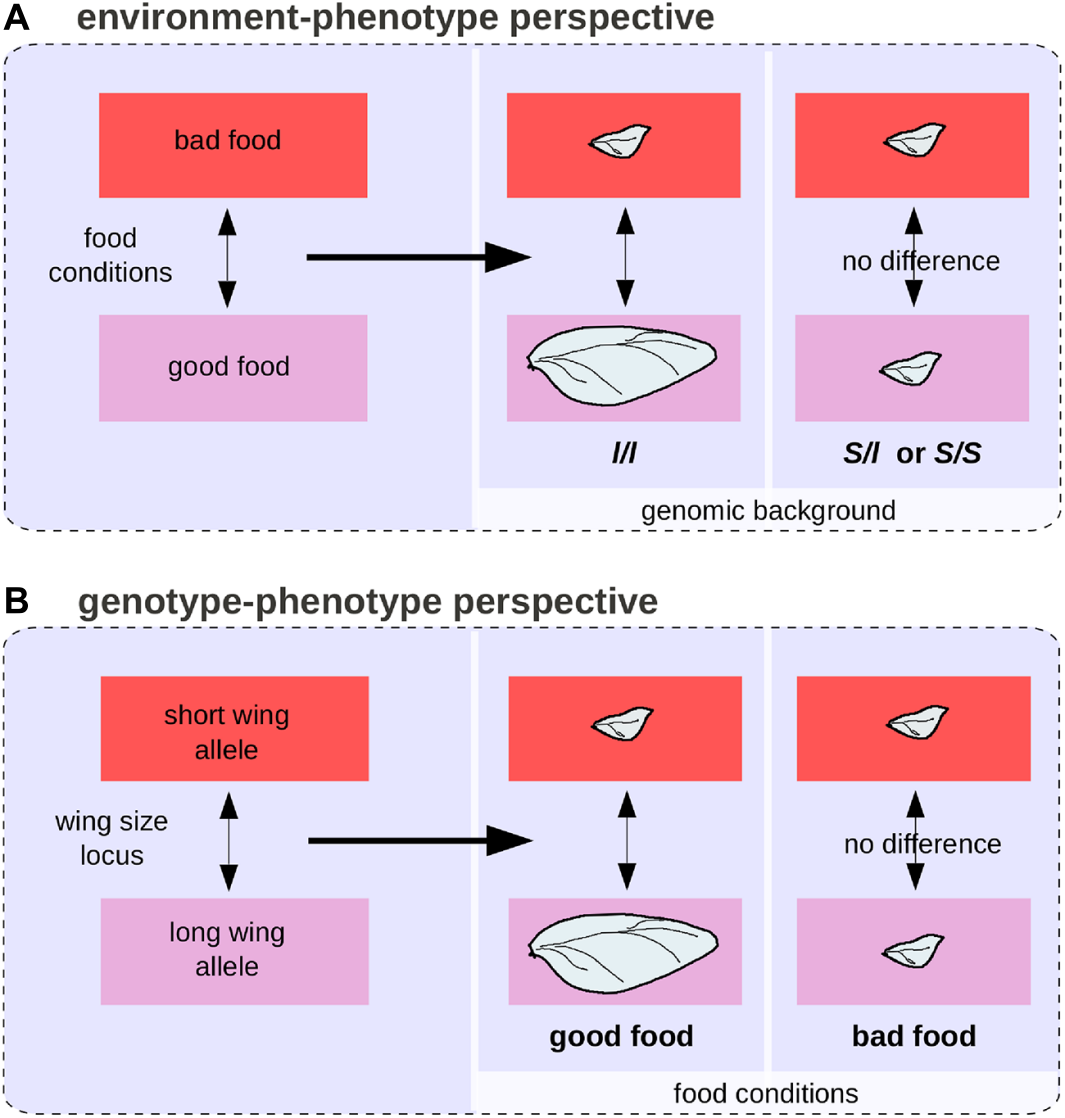
**The environment-phenotype relationship and GP relationship perspectives for wing length polymorphism in the beetle *Calathus melanocephalus*. (A)** Under the environment-phenotype relationship perspective, a change in food conditions is associated with a change in wing size, but only in a homozygous background for the recessive allele (*l*) of the wing size locus. **(B)** Under the GP relationship perspective, a genetic change at the wing size locus is associated with a change in wing size, but only in good food conditions.

Various quantitative methods have been developed to disentangle genetic from environmental effects and to quantify GxE interactions (Lynch and Walsh, 1998). Yet in certain situations it can be impossible to separate genetic from environmental effects in a biologically meaningful way, even when reasoning in terms of differences (Lewontin, 1974b). Populations of the beetle *Calathus melanocephalus* comprise two morphs, long-winged, and short-winged (Schwander and Leimar, 2011). The long-winged morph only develop from homozygous individuals for a recessive allele segregating in the population, and only when food conditions are good. In this case, the genetic and environmental effects are intermingled (**Figures 6A,B**). In the theoretical case of a population comprising only short-winged heterozygous animal that have been raised in starving conditions and long-winged ones, both genes and environment are responsible for the wing difference between individuals and it is impossible to estimate the proportion of environmental and genetic effects because genes and environment act on distinct levels along the complex causal link between genotypes and phenotypes.

Another case that questions the classical environment/genetic distinction is when the addition of certain symbiotic bacteria modifies the host phenotype. Mice fed with a *Lactobacillus* strain of bacteria show reduced anxiety-related behaviors compared to control mice fed with broth without bacteria (Bravo et al., 2011). Here the behavioral difference is caused by a switch between presence or absence of a particular gut symbiont. The cause of the phenotypic difference is not a simple change in a DNA sequence, nor a simple environmental change disconnected from genetic changes, but a switch between presence and absence of a factor that can be considered as an environmental factor – the bacteria – which contains DNA whose mutations may also change the host phenotype.

In conclusion, reasoning in terms of differences can help to clarify the comparison between genetic and environmental effects on phenotypes. However, the issues are nothing but simple. Since genes and environment act on distinct levels along the complex causal link between genotypes and phenotypes, in certain cases it is impossible to disentangle both causes.

## A Clarification on the Terminology Gain/Loss and Permissive/Instructive

Phenotypic differences appear to fall under two major categories, either the presence/absence of something (for example body hair or the ability to digest milk), or the shift between two alternatives that are both present (for example two hair colors). Similarly, on the genotype side, a mutation can correspond to the presence/absence of a relevant DNA sequence, or to a nucleotide polymorphism. The differential perspective makes it evident that a loss of phenotype is not necessarily associated with a loss of genetic material, and vice versa. For example, the evolutionary gain of dark pigments covering the entire coat of animals has often been associated with a loss of the *Mc1R* gene (Gompel and Prud’homme, 2009). Furthermore, as one of us noted previously (appendix of Stern and Orgogozo, 2008), gain or loss for a phenotype is subjective. For example, loss of hair might also be considered as gain of naked epidermis. Most insect epidermal cells differentiate into one of these two alternative states and both states involve large gene regulatory networks. It is not clear which phenotypic state represents a gain or loss relative to the other. Even on the genotypic side, defining losses and gains can be difficult. The insertion of a transposable element can knock down a gene, whereas a deletion can sometimes creates a new binding site for an activator of transcription. As a matter of fact, the evolutionary gain of *desatF* expression in *D. melanogaster* occurred through a series of three deletions, each creating an hexamer motif that is required for *desatF* expression (Shirangi et al., 2009).

Similarly, the differential perspective on environmental effects highlights the fallacy of the distinction between permissive and instructive signals. A permissive signal is associated with the presence/absence of a phenotype and an instructive signal with the shift between two alternatives that are both present. As argued above, these distinctions at the phenotypic level are not clear-cut.

In conclusion, we suggest that the gain/loss and instructive/permissive terminology should be used with caution.

## Taxonomically Robust GP Relationships

A mutation is expected to produce a somewhat reproducible phenotypic variation within a population. Such reproducibility in phenotypic outcome is required to allow genetic evolution and adaptation by natural selection (Lewontin, 1974a; Kirschner and Gerhart, 1998). Indeed, a newly formed allele that would generate yet another phenotype each time it ends up in a different organism would not be subjected to natural selection. Reasoning in terms of variation, rather than considering alleles as isolated entities, makes it clear that competition occurs between alleles that span the same genetic locus. Natural selection acts directly on the allelic variation that is consistently associated with a given phenotypic variation, which is the GP relationship itself. The GP relationship is thus a basic unit of evolutionary change, on which natural selection acts.

A major discovery of the past 20 years is that variation at certain genetic loci produce comparable phenotypic variation not only in various individuals of one population, but also in extremely diverse taxa (Martin and Orgogozo, 2013b). In other words, certain GP relationships are taxonomically robust and present across a large range of species. This implies that the genetic and environmental backgrounds have remained relatively constant or have appeared repeatedly throughout evolution to allow for genetic loci to generate similar phenotypic changes in various taxonomic groups. This important finding was quite unsuspected some 50 years ago. For a long time the singularity observed in the living world was expected to reflect a comparable singularity at the genetic level, implicating disparate and non-conserved genes, specific to each lineage (Mayr, 1963). As Mayr (1963) once proposed in 1963, “Much that has been learned about gene physiology makes it evident that the search for homologous genes is quite futile except in very close relatives […]. The saying “Many roads lead to Rome” is as true in evolution as in daily affairs” (Mayr, 1963). In other words, the genetic loci that make a man a man were expected to be different from the ones that make a dog, or a fish. Later, in the 80–90s, a few researchers suggested quite the contrary that evolution proceeds through mutations in conserved protein-coding genes (Romero-Herrera et al., 1978; Perutz, 1983; Stewart et al., 1987; Carroll et al., 2005) – but they had little experimental data at hand to support their view (Tautz and Schmid, 1998). As of today, the accumulating data on the mutations responsible for natural variation make it clear that the diversity in living organisms share a common genetic basis on at least three points. First, comparative developmental biology revealed that animals share common sets of key regulatory genes with conserved functions (Wilkins, 2002, 2014; Carroll et al., 2005). Second, most interspecific differences in animals and plants for which the underlying genetic basis has been at least partly identified (154 cases out of 160) are due to mutations at homologous genes, and very few (6/160) are due to new genes, which nevertheless represent duplicates of existing genes (Martin and Orgogozo, 2013b). Third, multiple cases of similar phenotypic changes have been shown to involve mutations of the same homologous genes in independent lineages (**Table 1**), sometimes across large phylogenetic distances. For instance, the difference in pigmentation between white and orange Bengal tigers has recently been mapped to a single mutation in the transporter protein gene *SLC45A2* (Xu et al., 2013), and this gene has also been associated with hypopigmented eyes, skin, hair, and feathers in humans and chickens (Xu et al., 2013; **Figure 1E**). A more dramatic example is the recent evolution of a toxin resistance in three species that diverged more than 500 million years ago – a clam, a snake and a pufferfish – via the same amino acid substitution in a conserved gene (Bricelj et al., 2005; Geffeney et al., 2005; Venkatesh et al., 2005; Feldman et al., 2012). Such striking patterns of genetic repetition have now been found for more than 100 genes in animals and plants (Martin and Orgogozo, 2013b). Despite existing methodological biases favoring conserved genes in the search for quantitative trait loci (Rockman, 2012; Martin and Orgogozo, 2013b), the level of genetic repetition remains astounding and suggests that for the evolution of at least certain phenotypic differences, relatively few genetic roads lead to Rome (Stern, 2013). Nowadays, one should not be surprised that a piece of DNA associated with a complex wing color pattern in one *Heliconius* butterfly species provides similar wings and collective protection from the same predators when introduced into the genome of other butterflies (Supple et al., 2014). What makes a dog a dog or a man a man is now partly explained by singular assortments of taxonomically robust GP relationships, which are found in multiple lineage branches.

Certain environment–phenotype relationships are also taxonomically robust. For example, across most taxa body size is affected by nutrition; iron deficiency can cause anemia and certain toxic compounds can be lethal. In ectotherms the temperature of the organism depends on the environmental temperature. Given the daunting number of environmental conditions that can be conceived, it is probably impossible to determine whether taxonomically robust GP relationships or taxonomically robust environment–phenotype relationships are more prevalent. Furthermore, whether taxonomically robust GP relationships represent an exceptional and small fraction, or a significant proportion, of all GP relationships is a matter of debate. In any case, the existence of taxonomically robust GP relationships is now clear and should be broadly accepted by the research community.

Some of the most striking teachings of modern biology include the discovery that living beings share the same genetic material (DNA or RNA), the same genetic code (with few exceptions), and the same basic cellular machinery. It is thus far from paradoxical that individual differences are built upon similarities, and the finding that certain GP relationships persists over long evolutionary times completes the picture.

The precise predictive power resulting from the existence of taxonomically robust GP relationships is rare in biology, and is only starting to be exploited at its full potential. Long-range conservations of GP links now fully justify the use of comparative genetics approaches to tackle pragmatic problems. For instance, crop domestication took the form of similar selective pressures in many species, and we now have experimental evidence that this process has repeatedly involved mutations in the same set of conserved genes (Paterson et al., 1995; Martin and Orgogozo, 2013b). This observation opens up interesting applications, as we can use this emerging body of genetic expertise to assist the domestication of future crops, or to use marker-assisted strategies to produce and maintain crop biodiversity (Lenser and Theißen, 2013). GP predictability is already used in the identification of strains that evolved resistance to different pest control strategies, with extreme cases targeting anti-malarial drugs tolerance in *Plasmodium* parasites (Manske et al., 2012), antibiotic resistance in bacteria and yeasts (Fischbach, 2009; MacCallum et al., 2010), or even more dramatically, the anthropogenic evolution of insecticide-resistance in diverse cohorts of insects, regardless of their pest status (Ffrench-Constant et al., 2004; Martin and Orgogozo, 2013b).

Furthermore, repeatability in the genetic basis of phenotypic variation suggests that clinical research is also likely to benefit from genetic studies of a large range of model species (Robinson and Webber, 2014). For instance, natural variation in the tolerance to methotrexate, a chemotherapy drug, was mapped in *Drosophila* fruitflies to genes whose human orthologs are also associated with the response of patients to this drug (Kislukhin et al., 2013), thus extending the use of model organisms as disease models.

## Toward a Gene-Based Classification of Phenotypes

One original aspect of framing the GP connection in terms of individual GP relationships is that it allows to classify phenotypes according to their underlying genetic basis. On a first level, GP relationships implicating different regions within the same gene and producing comparable phenotypic outcomes can be grouped together. Simple cases of GxG interactions have been found between tightly linked mutations, generally within a coding sequence or within a *cis-*regulatory element, when they generate a non-additive effect on the phenotype. For example, a particular mutation in an enhancer was observed to produce different shifts in expression pattern of the downstream coding gene, depending on neighboring DNA sequence (Frankel et al., 2011; Rogers et al., 2013). Similarly, amino acid mutations in a hemoglobin gene was found to increase or decrease affinity to oxygen, depending on the allelic state of other sites (Natarajan et al., 2013). In such cases, it is intuitive to group such genetically linked sites together as they all affect the same kind of phenotypic trait.

Absence of melanin pigments in animals has been associated with mutations in several genes, including *OCA2*, *kit ligand* or *Mc1R* (reviewed in Gompel and Prud’homme, 2009; Liu et al., 2013). Whereas the absence of melanin is traditionally considered as one character state, albinism, irrespective of the underlying genetic basis, we propose here to distinguish *OCA2*-associated albinism from *Mc1R*-associated albinism, or from albinism associated with any other gene. One interest of decomposing the variation within the living world into these multiple elementary GP relationships is that these elements can then be grouped together into successively larger groups. Elementary phenotypic changes involving different genes that are part of the same genetic pathway could also be grouped together as concomitant components of the same phenotype-modulating mechanism. This is clearly the case for the TGF-β signaling molecules BMP15, GDF9, and the TGF-β receptor BMPR1B, that have all been repeatedly associated to variations in ovarian function in humans and in domestic sheep breeds (reviewed in Luong et al., 2011).

Another important consequence of the GP relationship perspective is that apparently distinct phenotypic changes caused by similar genetic loci in various organisms can be examined further to uncover what might be a common basic phenotypic change (Deans et al., 2015). For example, fly larvae and nematode worms have distinctive food search behaviors but mutations in the same orthologous gene (*for*/*egl-4*) have been shown to alter the intensity of food search behaviors in both organisms (Osborne et al., 1997; Mery et al., 2007; Hong et al., 2008). It is thus plausible that a basic behavioral change, which underlies seemingly distinctive fly and nematode food search changes, represents a conserved GP relationship across nematodes and flies. This somewhat borderline example illustrates the challenge to incorporate widespread comparative thinking into our global understanding of biology. Is a mutation in a mouse model relevant to human disease? Can we consider that a mouse phenotype is similar to a human condition if its genetic basis is different? We and others predict that the search for orthologous phenotypes, or “phenologs” (McGary et al., 2010), will represent a major task for modern genetics and will require a fruitful alliance between applied and evolutionary biology.

## Conclusion

In this paper, we bring back the differential concept of gene (Schwartz, 2000) into our framework for understanding the GP map. The differential view of the GP relationship helps to clarify the genetic and environmental effects on phenotypes and their connection. It also opens up new avenues of thinking, in particular regarding the decomposition of observable features within an organism and the representation of GP maps. Furthermore, the existence of taxonomically robust GP relationships encourages an unabashed use of comparative genetics to predict the genetic basis of phenotypic variation in diverse groups of organisms, and this predictive power has an important potential for translational research in agronomy and clinical research.

## Conflict of Interest Statement

The authors declare that the research was conducted in the absence of any commercial or financial relationships that could be construed as a potential conflict of interest.

## Abbreviations

GP: genotype–phenotype.
